# Queen Charlotte's Lying-In Hospital

**Published:** 1898-09-17

**Authors:** 


### Editor's Letter-Box.

[Our Correspondents are reminded that prolixity is a great bar to publication, and that brevity of style and conciseness of statement greatly facilitate early insertion.]

### QUEEN CHARLOTTE'S LYING-IN HOSPITAL.

Mr. Arthur Walls, secretary to the Queen Charlotte's Lying-in Hospital, writes to draw attention to the facilities offered to students of midwifery by that institution. The men do all their work within the walls of the hospital, near to which they must reside during the time occupied by the course. They must not attend any medical or surgical cases outside the hospital, nor any post-mortem examination. The period of attendance is for not less than four weeks.

